# Extended-spectrum beta-lactamase phenotype in *Klebsiella pneumoniae* isolates from Meta Department hospitals, Colombia, 2018–2022: A retrospective analytical cross-sectional study

**DOI:** 10.1371/journal.pone.0352453

**Published:** 2026-08-04

**Authors:** Nancy Giovanna Cocunubo-Cocunubo, Norma Pavas-Escobar, Liliana Marcela Montilla Rodríguez, Fabiana Soto-Gutiérrez, Angie K. Rojas-Zapata, Laura Sánchez-Pabón, Laura Melissa López-Rincón, Norton Pérez-Gutiérrez

**Affiliations:** 1 Universidad Cooperativa de Colombia, Facultad de Medicina, Villavicencio, Colombia; 2 Grupo de investigación de Villavicencio - GRIVI, Villavicencio, Colombia; 3 Secretaría de Salud del Meta, Laboratorio de Salud Pública, GISSME, Villavicencio, Colombia; 4 Hospital Departamental de Villavicencio, Villavicencio, Colombia; Rivers State University, NIGERIA

## Abstract

**Background:**

Antimicrobial resistance (AMR) is a major global health concern. *Klebsiella pneumoniae* is a key pathogen in healthcare-associated infections due to the production of extended-spectrum beta-lactamases (ESBLs), which limit therapeutic options.

**Objective:**

To estimate the prevalence and identify factors associated with the presumptive ESBL phenotype in *K. pneumoniae* isolates from hospitals in the Meta Department, Colombia, between 2018 and 2022.

**Methods:**

We conducted a retrospective analytical cross-sectional study using 4,808 non-carbapenem-resistant *K. pneumoniae* isolates obtained from routine microbiological surveillance. Bivariate analyses (Chi-square and Mann–Whitney U tests) and multivariable logistic regression were performed to evaluate associations between ESBL production and demographic, clinical, and institutional variables.

**Results:**

A total of 4,808 non-carbapenem-resistant *Klebsiella pneumoniae* isolates were included after applying exclusion criteria. The overall prevalence of the presumptive ESBL phenotype was 22.9% (n = 1,100). In bivariate analysis, ESBL phenotype was significantly associated with male sex, older age, inpatient care, hospital type, and specimen type, particularly urine and blood samples. In multivariable analysis, male sex, inpatient status, and urine specimens remained independently associated, along with variability across specific hospitals, indicating the influence of institutional factors. The model showed limited discriminative ability (AUC = 0.62) and low explanatory power (pseudo-R² 0.03–0.05).

**Conclusions:**

The presumptive ESBL phenotype in *Klebsiella pneumoniae* is highly prevalent and increasing over time. Its association with demographic, clinical, and institutional factors highlights the multifactorial nature of antimicrobial resistance and supports the need for strengthened surveillance and targeted antimicrobial stewardship strategies.

## 1. Introduction

Antimicrobial resistance (AMR) is recognized as one of the most critical global public health challenges, as it contributes to increased morbidity and mortality, prolonged hospital stays, and a substantial rise in healthcare costs [1,2]. The rapid emergence and dissemination of resistant bacterial pathogens have led international organizations, including the World Health Organization (WHO), to recommend the integration of AMR surveillance as a central component of antimicrobial stewardship programs (ASPs), aimed at promoting rational antibiotic use and preserving the effectiveness of existing therapies [3,4].

*Klebsiella pneumoniae* is a clinically significant opportunistic Gram-negative pathogen and a major cause of healthcare-associated infections (HAIs), including pneumonia, bloodstream infections, urinary tract infections, and intra-abdominal infections [5]. It’s remarkable capacity to acquire and disseminate resistance determinants through mobile genetic elements has positioned *K. pneumoniae* as a key member of the ESKAPE group of pathogens, which are responsible for a substantial proportion of multidrug-resistant infections worldwide [6].

A major concern is the increasing prevalence of resistance to third-generation cephalosporins, commonly associated with the presence of extended-spectrum beta-lactamases (ESBLs). These enzymes confer resistance to a broad range of beta-lactam antibiotics, significantly limiting therapeutic options and often leading to increased reliance on carbapenems, thereby accelerating the emergence of resistance to last-line agents [7,8]. Infections caused by Enterobacterales exhibiting ESBL-associated resistance phenotypes have been associated with delays in appropriate antimicrobial therapy, increased mortality, and higher healthcare costs [9].

In low- and middle-income countries (LMICs), including Colombia, *K. pneumoniae* with ESBL-associated resistance phenotypes represents a major cause of HAIs, particularly in intensive care units (ICUs), where high antibiotic pressure, invasive procedures, and prolonged hospitalization favor the selection and transmission of resistant strains [10]. Surveillance data at the national level have consistently reported high resistance rates to third-generation cephalosporins in *K. pneumoniae*, with similar patterns observed across different hospital settings. In this context, resistance to these antibiotics is frequently used as a phenotypic screening approach for identifying presumptive ESBL phenotype, although confirmatory testing based on clavulanate inhibition is not always available [11].

Despite the availability of national surveillance data, substantial heterogeneity persists in antimicrobial resistance patterns across regions, healthcare institutions, and clinical settings. Variability in geographic, demographic, and healthcare system characteristics within Colombia may significantly influence local resistance dynamics, thereby limiting the applicability of aggregated national estimates for guiding empirical antibiotic therapy at the regional level. Recent evidence indicates that resistance profiles may vary considerably even within the same country, underscoring the need for region-specific analyses to inform clinical decision-making and public health interventions [12,13].

The Meta Department, located in the Colombian Orinoquía region, presents distinct epidemiological and healthcare characteristics; however, data on the burden and determinants of resistance phenotypes compatible with ESBL in *K. pneumoniae* remain limited. Strengthening regional surveillance is essential to support evidence-based empirical treatment guidelines, optimize antimicrobial stewardship strategies, and inform infection prevention and control policies.

Therefore, the aim of this study was to estimate the prevalence and identify independent factors associated with the presumptive ESBL phenotype in *Klebsiella pneumoniae* isolates collected from hospitals in the Meta Department, Colombia, between 2018 and 2022, using an analytical cross-sectional design based on retrospectively collected surveillance data

## 2. Materials and methods

### 2.1. Study design and data source

An analytical cross-sectional study based on retrospectively collected surveillance data was conducted using a secondary database derived from routine microbiological surveillance. The dataset included records from 11 hospitals in the Meta Department, Colombia, covering the period from January 2018 to December 2022. Within the framework of the National Antimicrobial Resistance Surveillance Program coordinated by the Colombian National Institute of Health, all participating institutions systematically reported microbiological data monthly to the Departmental Public Health Laboratory. This surveillance system operates under standardized national protocols for data collection, validation, and reporting. No direct involvement of human participants occurred, as the study was based exclusively on anonymized secondary data.

### 2.2. Microbiological methods and ESBL definition

All participating hospitals employed automated systems for bacterial identification and antimicrobial susceptibility testing (AST), such as Vitek® or Phoenix®, in accordance with each laboratory’s standard operating procedures. Susceptibility results were interpreted following the Clinical and Laboratory Standards Institute (CLSI) guidelines applicable to each year of the surveillance period.

For epidemiological surveillance purposes, a presumptive ESBL phenotype was defined based on phenotypic screening criteria, as confirmatory clavulanate inhibition tests were not uniformly available across all participating institutions. According to the Colombian National Institute of Health, microbiological reports used for antimicrobial resistance surveillance were standardized prior to their incorporation into the regional surveillance system [14]. This approach is consistent with antimicrobial resistance surveillance systems in low- and middle-income countries and aligns with the methodological framework recommended by the World Health Organization through the Global Antimicrobial Resistance Surveillance System (GLASS), ensuring international comparability [15–17].

In this study, *Klebsiella pneumoniae* isolates were classified as exhibiting a presumptive ESBL phenotype if they showed resistance to ceftazidime (CAZ; minimum inhibitory concentration [MIC] > 4 μg/mL) and/or cefotaxime (CTX; MIC > 1 μg/mL), in accordance with INS protocols and CLSI guidelines. This definition reflects a screening-based phenotypic approach and does not confirm ESBL production; therefore, it may include other resistance mechanisms, such as AmpC β-lactamases. To reduce misclassification and avoid the inclusion of carbapenem-resistant Enterobacterales (CRE), isolates showing resistance to any tested carbapenem (ertapenem, imipenem, or meropenem) were excluded from the analysis.

### 2.3. Study population and inclusion criteria

The surveillance database contained information on the date of isolation, the type and level of the healthcare facility, the hospital ward (including intensive care unit [ICU], inpatient ward, emergency department, or outpatient setting), the type of biological specimen (as reported by the laboratory), bacterial identification, and antimicrobial resistance profile. Initially, all microbiological isolation records from hospitals in Villavicencio and Granada (Meta Department, Colombia) during the study period were considered.

The original database included patient-level identifiers (internal codes, national identification numbers, and names), which allowed the identification of multiple records from the same individual. Prior to analysis, the dataset was reviewed and deduplicated to ensure that only one isolate per patient was included. Subsequently, all personal identifiers were removed to guarantee data anonymization and compliance with ethical standards.

For the final analysis, only records corresponding to *Klebsiella pneumoniae* isolates were included. Records were excluded if they showed documented carbapenem resistance, corresponded to laboratory quality control procedures, or lacked complete information on bacterial identification or antimicrobial susceptibility results.

The primary outcome was the presumptive ESBL phenotype, defined phenotypically as resistance to ceftazidime (CAZ) and/or cefotaxime (CTX), based on established minimum inhibitory concentration (MIC) cutoff values. This definition corresponds to a screening-based criterion derived from microbiological surveillance data and does not imply confirmed ESBL production.

### 2.4. Data analysis

Categorical variables were summarized using absolute frequencies and proportions. Quantitative variables were assessed for distribution and described using the median and interquartile range (IQR) or the mean and standard deviation, as appropriate. Data were exported to Microsoft Excel and analyzed using Prism software (GraphPad, version 10.0.0 for Mac/iOS, Boston, Massachusetts, USA).

Bivariate analyses were performed to evaluate associations between independent variables (age, sex, hospital type, level of care, hospital ward, and specimen type) and the outcome of interest, defined as the presumptive ESBL phenotype. Differences between categorical variables were assessed using the chi-square (χ²) test or Fisher’s exact test, as appropriate. Quantitative variables (e.g., age) were compared using the Mann–Whitney U test due to non-normal distribution. Measures of association were reported as odds ratios (ORs) with 95% confidence intervals (95% CIs). A p-value < 0.05 was considered statistically significant.

Subsequently, a multivariable logistic regression model was constructed to identify independent factors associated with the presumptive ESBL phenotype. Given the structure of the available surveillance data, variables included in the model were selected based on both statistical criteria (p < 0.20 in bivariate analysis) and epidemiological relevance. Although clinically relevant variables such as prior antibiotic use, comorbidities, and length of hospital stay were not available in the dataset, the model was designed to evaluate structural and demographic factors associated with the outcome. Model discrimination was assessed using the area under the receiver operating characteristic curve (AUC), and explanatory capacity was evaluated using Cox & Snell and Nagelkerke pseudo-R² statistics

### 2.5. Ethical considerations

The present study was based exclusively on the analysis of secondary data derived from routine microbiological surveillance systems in public health, systematically collected by participating institutions under previously established institutional and national protocols. There was no direct involvement of human participants, and no biological samples were collected for research purposes. The study protocol was reviewed by the Bioethics Subcommittee of the Universidad Cooperativa de Colombia (Villavicencio Campus), which determined that the research met the criteria for exemption from ethical approval, as it involved the use of fully anonymized secondary data and posed no risk to individuals. Accordingly, the study was classified as research without risk, and a formal waiver of ethical approval was granted. The database was anonymized by the corresponding public health authority prior to access by the investigators, with all information that could allow direct or indirect identification of individuals removed. At no point during or after data access did the authors have access to identifiable information. The study was conducted in accordance with national regulations for health research (Resolution 8430 of 1993) [18] and international ethical standards, including the Declaration of Helsinki [19] and the guidelines of the Council for International Organizations of Medical Sciences (CIOMS) in collaboration with the World Health Organization (WHO) [20]. To ensure institutional confidentiality, each participating hospital was assigned a letter of the alphabet (A–K). The quality of reporting was assessed using the STROBE checklist.

## 3. Results

### 3.1. Flowchart of selection, exclusion, and classification of *Klebsiella pneumoniae* isolates according to presumptive extended-spectrum β-lactamase (ESBL) production

**Fig 1:**
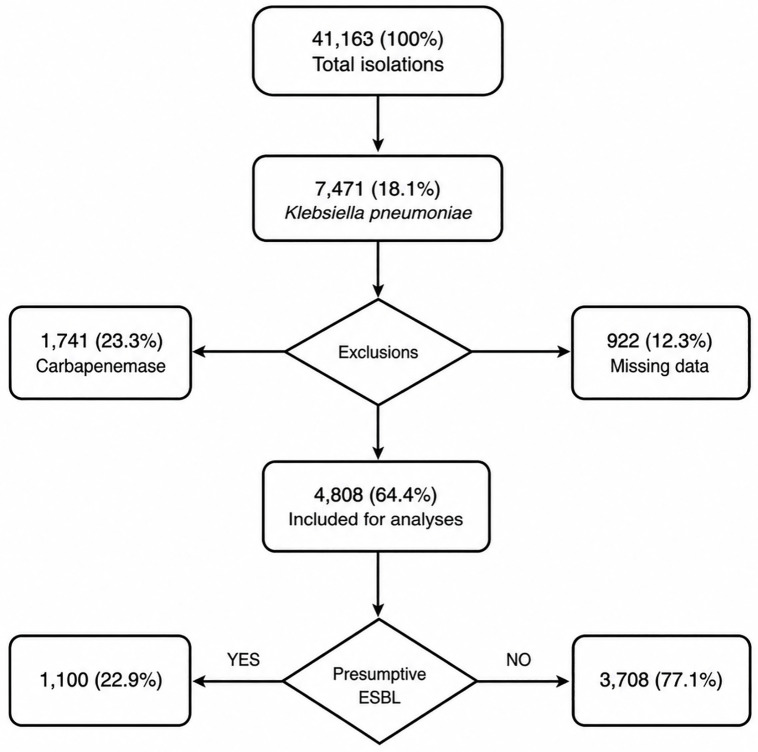
Flowchart of *Klebsiella pneumoniae* isolate selection for the study. Flowchart showing the selection of *Klebsiella pneumoniae* isolates included in the study and the application of exclusion criteria, resulting in the final analytical dataset and classification according to presumptive ESBL phenotype. ESBL = extended-spectrum beta-lactamase. Source**:** Original from the authors.

As shown in Fig 1, a total of 41,163 bacterial isolates (100%) were initially recorded, of which 7,471 (18.1%) corresponded to *Klebsiella pneumoniae*. Subsequently, exclusion criteria were applied to ensure data quality and analytical consistency. A total of 1,741 isolates (23.3%) identified as carbapenemase-producing strains were excluded due to their distinct resistance profile, along with 922 records (12.3%) due to missing or incomplete data. After these exclusions, 4,808 isolates (64.4%) were included in the final analytical dataset. Within this cohort, isolates were classified according to a presumptive extended-spectrum β-lactamase (ESBL) phenotype based on phenotypic screening criteria. Overall, 1,100 isolates (22.9%) were classified as presumptive ESBL producers, whereas 3,708 (77.1%) were categorized as non-ESBL.

### 3.2. Descriptive characteristics of presumptive ESBL-producing *Klebsiella pneumoniae* isolates

A total of 4,808 *Klebsiella pneumoniae* isolates were analyzed, of which 1,100 (22.9%) were classified as presumptive extended-spectrum β-lactamase (ESBL) producers and 3,708 (77.1%) as non-ESBL (Table 1). Regarding demographic characteristics, male patients predominated (56.1%) compared to females (43.9%). The median age was 61 years (IQR: 43–73), with a slightly higher median observed among ESBL cases (63 years) compared to non-ESBL isolates. In terms of patient type, most isolates corresponded to adults (92.0%), followed by geriatric patients (42.8%), while pediatric (6.4%) and neonatal (1.6%) groups represented smaller proportions. With respect to institutional origin, most isolates were obtained from private hospitals (62.6%), compared to public hospitals (37.4%). At the institutional level, hospitals D (30.6%), F (30.5%), and J (23.7%) accounted for the highest proportion of isolates. According to hospital service, isolates were primarily obtained from inpatient wards (36.2%), followed by intensive care units (30.8%) and emergency departments (29.3%), while the outpatient setting represented a smaller proportion (3.7%). Regarding specimen type, the most frequent sources were urine (33.3%), tracheal secretions (20.3%), and blood (17.5%), followed by other secretions (9.2%) and sputum (5.9%). Finally, the temporal distribution showed a higher frequency of isolates in 2021 (27.8%), followed by 2019 (21.3%), 2020 (19.2%), 2022 (16.2%), and 2018 (15.5%).

**Table 1:** Descriptive characteristics and bivariate analysis of factors associated with presumptive ESBL production in *Klebsiella pneumoniae* isolates from hospitals in the Meta Department, 2018–2022.

Variables	Total ESBL cases included in the analysis (n = 4808)		Descriptive characteristics of presumptive ESBL-producing *Klebsiella pneumoniae* isolates, 2018–2022 (n = 4808)				Bivariate analysis of the association between selected variables and presumptive ESBL production in *Klebsiella pneumoniae* (n = 4808)			
							OR value	95% Confidence Interval		P-value
	n	%	ESBL	%	No ESBL	%		Lower	Upper	
Isolates included for analyses	4808	100.0	1100	22.9	3708	77.1				
Gender										
Male	2698	56.1	673	24.9	2025	75.1	1.31	1.14	1.50	< 0.001
Female	2110	43.9	427	20.2	1683	79.8	–	–	–	–
Age (years, median, IQR)	61	(43-73)	63	(44-73)	61	(61-73)				0.03
Type of patient										
Newborn	78	1.6	13	16.7	65	83.3	–	–	–	–
Pediatric	309	6.4	81	26.2	228	73.8	1.78	0.93	3.39	0.08
Adult	4421	92.0	1006	22.8	3415	77.2	1.47	0.81	2.68	0.20
Geriatrics	2058	42.8	518	25.2	1540	74.8	1.68	0.92	3.08	0.09
Hospital										
A	86	1.8	29	33.7	57	66.3	2.59	1.52	4.42	< 0.001
B	108	2.2	26	24.1	82	75.9	1.61	0.94	2.73	0.07
C	36	0.7	13	36.1	23	63.9	2.88	1.37	6.03	< 0.001
D	1471	30.6	316	21.5	1155	78.5	1.39	1.01	1.91	0.04
E	329	6.8	54	16.4	275	83.6	–	–	–	–
F	1467	30.5	273	18.6	1194	81.4	1.16	0.85	1.60	0.35
G	54	1.1	4	7.4	50	92.6	0.41	0.14	1.18	0.09
I	115	2.4	43	37.4	72	62.6	3,04	1.89	4.90	< 0.001
J	1140	23.7	342	30.0	798	70.0	2,18	1.59	3.00	< 0.001
K	2	0.0	0	0.0	2	100.0	–	–	–	–
Private hospital	3012	62.6	773	25.7	2239	74.3	1.55	1.34	1.79	< 0.001
Public hospital	1796	37.4	327	18,2	1469	81.8	–	–	–	–
Ward type										
Emergency room	1411	29.3	324	23.0	1087	77.0	1.40	0.93	2.11	0.10
ICU	1481	30.8	261	17.6	1220	82.4	1.01	0.67	1.52	0.97
Inpatient	1739	36.2	484	27.8	1255	72.2	1.82	1.22	2.71	< 0.001
Ambulatory	177	3.7	31	17.5	146	82.5	–	–	–	–
Specimen type										
Urine	1599	33.3	434	27.1	1165	72.9	2.56	1.77	3.69	< 0.001
Tracheal secretion	977	20.3	128	13.1	849	86.9	1.03	0.70	0154	0.87
Blood	843	17.5	219	26.0	724	85.9	2.08	1.42	3.04	< 0.001
Secretion	441	9.2	131	29.7	310	70.3	0.80	0.49	1.30	0.36
Sputum	283	5.9	36	12.7	247	87.3	–	–	–	–
Year										
2018	745	15.5	166	22.3	579	77.7	1.44	1.15	1.80	< 0.001
2019	1025	21.3	227	22.1	798	77.9	1.43	1.16	1.75	< 0.001
2020	921	19.2	223	24.2	698	75.8	1.60	1.30	1.97	< 0.001
2021	1336	27.8	222	16.6	1114	83.4	–	–	–	–
2022	781	16.2	262	33.5	519	66.5	2.53	2.06	3.12	< 0.001

ESBL = extended-spectrum beta-lactamase.

Source: Original from the authors.

### 3.3. Bivariate analysis of the association between selected variables and presumptive ESBL production in *Klebsiella pneumoniae*

**Fig 2:**
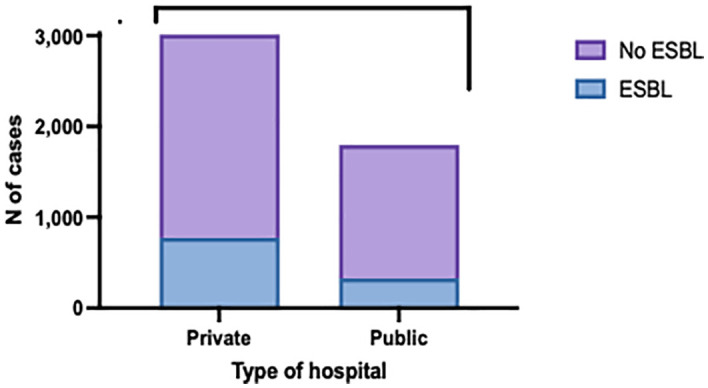
ESBL frequency according to hospital type in Meta Department hospitals. The figure compares the proportion of presumptive ESBL-producing *Klebsiella pneumoniae* isolates between public and private hospitals during 2018–2022. Bars represent percentages of ESBL-positive isolates in each hospital type. ESBL = extended-spectrum beta-lactamase. Source: Original from the authors.

**Fig 3:**
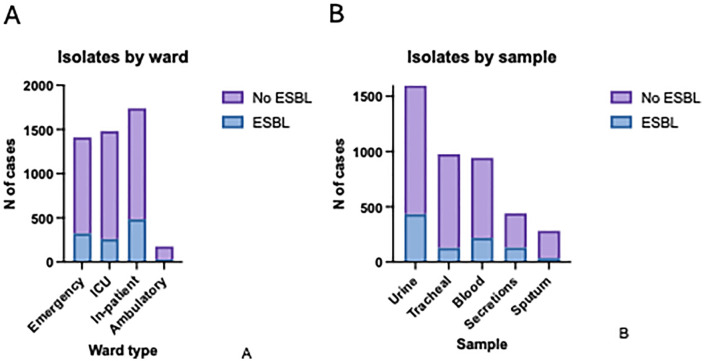
Distribution of *Klebsiella pneumoniae* isolates by ward and clinical specimen. (A) Distribution of *Klebsiella pneumoniae* isolates according to hospital ward. (B) Distribution according to clinical specimen. Bars show the total number of isolates and the proportion of presumptive ESBL-producing isolates in each category. ESBL = extended-spectrum beta-lactamase. Source: Original from the authors.

As shown in Table 1, the bivariate analysis identified multiple variables associated with presumptive extended-spectrum β-lactamase (ESBL) production in *Klebsiella pneumoniae* isolates. Regarding demographic characteristics, male sex was associated with a higher likelihood of ESBL production compared to females (OR = 1.31; 95% CI: 1.14–1.50; p < 0.001). A significant association was also observed with age (p = 0.03), with a higher median age among ESBL cases. No statistically significant associations were identified according to patient type (p > 0.05), although higher proportions were observed in pediatric and geriatric groups. With respect to the institution of origin, heterogeneity was observed across hospitals. Significant associations were identified in hospitals A (OR = 2.59; 95% CI: 1.52–4.42; p < 0.001), C (OR = 2.88; 95% CI: 1.37–6.03; p < 0.001), D (OR = 1.39; 95% CI: 1.01–1.91; p = 0.04), I (OR = 3.04; 95% CI: 1.89–4.90; p < 0.001), and J (OR = 2.18; 95% CI: 1.59–3.00; p < 0.001). At an aggregate level, isolates from private hospitals showed higher odds of ESBL production compared to public hospitals (OR = 1.55; 95% CI: 1.34–1.79; p < 0.001), as illustrated in Fig 2. Regarding hospital service, isolates from inpatient settings were significantly associated with ESBL production (OR = 1.82; 95% CI: 1.22–2.71; p < 0.001), whereas no significant differences were observed for ICU or emergency departments. This pattern is shown in Fig 3A. In relation to specimen type, significant associations were identified for urine (OR = 2.56; 95% CI: 1.77–3.69; p < 0.001) and blood (OR = 2.08; 95% CI: 1.42–3.04; p < 0.001), as presented in Fig 3B. Finally, the temporal analysis showed significant associations in 2018, 2019, 2020, and 2022, with 2022 showing the highest magnitude (OR = 2.53; 95% CI: 2.06–3.12; p < 0.001) compared to 2021 as the reference category. The bivariate analysis demonstrates that presumptive ESBL production in *K. pneumoniae* is significantly associated with demographic, institutional, clinical, and microbiological factors, showing patterns consistent with those observed in Figs 2 and 3, particularly in relation to hospital type, care setting, and specimen type.

### 3.4. Multivariable logistic regression analysis of factors associated with presumptive ESBL production in *Klebsiella pneumoniae* isolates

**Fig 4:**
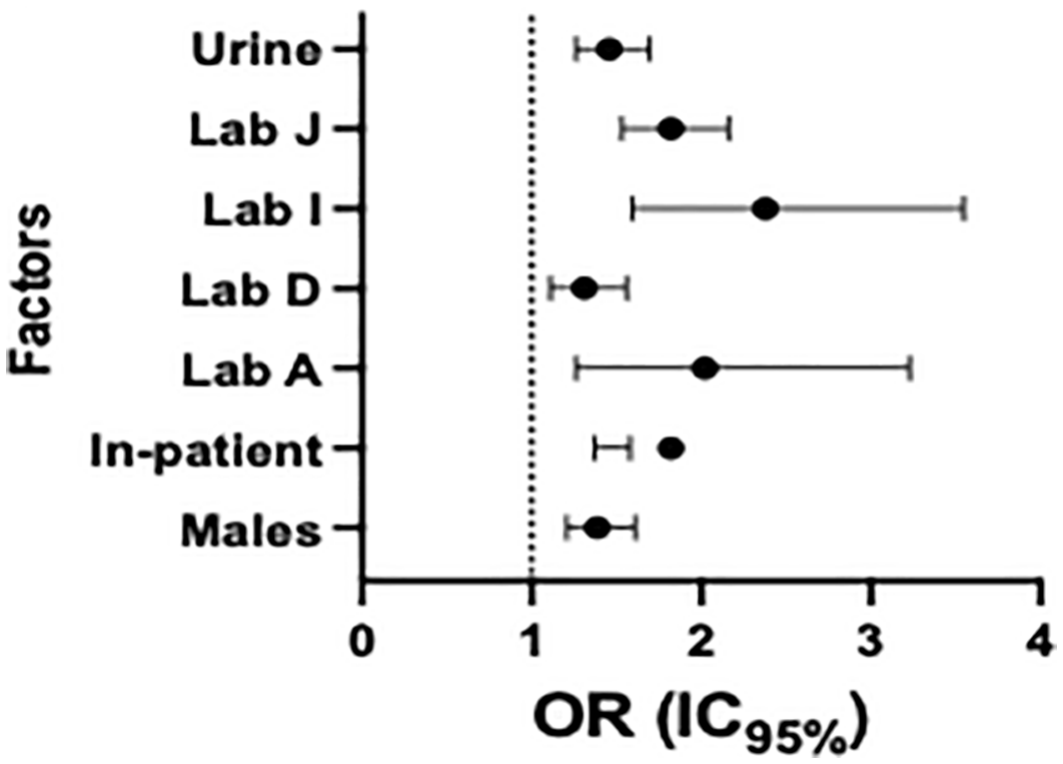
Factors associated with presumptive ESBL production in *Klebsiella pneumoniae.* Forest plot showing adjusted odds ratios (ORs) and 95% confidence intervals obtained from the multivariable logistic regression model evaluating factors associated with presumptive ESBL production. Variables with confidence intervals not crossing the null value indicate statistically significant associations. ESBL = extended-spectrum beta-lactamase; OR = odds ratio; CI = confidence interval. Source: Original from the authors.

As shown in Table 2 and Fig 4, a multivariable logistic regression model was developed to identify independent factors associated with presumptive extended-spectrum β-lactamase (ESBL) production in *Klebsiella pneumoniae* isolates. Variables were selected based on statistical criteria (p < 0.20 in the bivariate analysis) and epidemiological relevance. For parsimony, only variables that remained statistically significant after adjustment are presented. After adjustment, male sex, inpatient status, and urine specimens remained independently associated with ESBL production. At the institutional level, specific hospitals (A, D, I, and J) also showed statistically significant associations, indicating variability across centers. These factors were associated with increased odds of ESBL production, with adjusted odds ratios greater than 1 and 95% confidence intervals that did not cross unity. The model showed an area under the curve (AUC) of 0.62, indicating limited discriminative ability. In addition, the model demonstrated modest explanatory power, with a Cox & Snell pseudo-R² of 0.0302 and a Nagelkerke pseudo-R² of 0.0458, indicating that the variables included explained approximately 3% to 5% of the variability in the outcome.

**Table 2:** Multivariable logistic regression analysis of factors associated with ESBL production in *Klebsiella pneumoniae* isolates from the Meta Department hospital network, 2018–2022.

Variable	OR value	95% Confidence Interval		P-value
		Lower	Upper	
Male	1.39	1.21	1.61	<0.001
Inpatient	1.58	1.37	1.70	<0.001
Lab A	2.02	1.26	3.23	<0.001
Lab D	1.31	1.11	1.56	<0.001
Lab I	2.38	1.59	3.55	<0.001
Lab J	1.82	1.53	2.16	<0.001
Urine	1.46	1.26	1.70	<0.001

ESBL = extended-spectrum beta-lactamase.

Source: Original from the authors.

## 4. Discussion

This retrospective analysis of over 7,000 *Klebsiella pneumoniae* isolates provides robust epidemiological evidence of a concerning increase in the presumptive extended-spectrum β-lactamase (ESBL) phenotype in the Meta Department, Colombia. The observed prevalence (22.9%) is consistent with reports from Latin American antimicrobial resistance surveillance networks, which have documented a sustained rise in resistance to third-generation cephalosporins among Enterobacterales [21–24]. Compared with local data from approximately two decades ago reporting resistance rates around 12.5%, our findings suggest a substantial increase, highlighting persistent selective pressure and the limited effectiveness of current containment strategies [25].

A notable finding is the marked increase in the presumptive ESBL phenotype in 2022 (33.5%), following a transient decline in 2021. This pattern aligns with global evidence describing a post-pandemic rebound in antimicrobial resistance, likely driven by the widespread use of broad-spectrum antibiotics during the COVID-19 pandemic, which may have intensified selective pressure on bacterial populations [26,27]. These trends underscore the need to reassess empiric treatment strategies and reinforce antimicrobial stewardship programs, particularly in low- and middle-income countries where regulatory and surveillance capacities may be heterogeneous [21].

In the multivariable analysis, male sex, inpatient status, and urine specimens remained independently associated with the presumptive ESBL phenotype. Additionally, variability across hospitals suggests that institutional-level factors, such as infection control practices, antimicrobial prescribing patterns, and diagnostic capacity, may play a relevant role in shaping resistance dynamics. Importantly, variables that appeared significant in the bivariate analysis, such as hospital ward, did not retain statistical significance after adjustment, indicating that these associations were likely confounded by other factors rather than reflecting an independent effect. This finding reinforces the importance of multivariable modeling to disentangle complex epidemiological relationships [28]

The higher prevalence observed in general wards in the bivariate analysis (27.8% vs. ICU 17.6%) should therefore be interpreted with caution, as this association was not maintained after adjustment. This suggests that the observed differences are likely explained by confounding factors rather than by the ward itself, including variations in patient characteristics, sampling practices, and exposure to healthcare interventions.

Furthermore, the association between the presumptive ESBL phenotype and older age groups supports the hypothesis that healthcare exposure and cumulative antibiotic use contribute to the maintenance of resistant reservoirs, as previously described in international studies [13,29]. Older adults are frequently identified as key reservoirs for ESBL-producing Enterobacterales due to repeated contact with healthcare systems, higher comorbidity burden, and increased antibiotic exposure [30]. These findings support targeted infection prevention strategies, including risk-based screening and strengthened surveillance in high-risk populations [31–33].

The analysis by specimen type further highlights the role of invasive devices and urinary tract infections in the epidemiology of antimicrobial resistance [30]. Similar patterns have been observed in multicenter studies from Europe and North America, where catheter-associated biofilms act as persistent reservoirs for ESBL-producing *K. pneumoniae* and facilitate intra-hospital spread [34].The high frequency observed in catheter-related samples (51.0%) and urine (27.3%) underscores the importance of “device stewardship,” with protocols emphasizing timely removal of catheters to reduce biofilm-related colonization, as recommended by international guidelines [35,36]. Additionally, the detection of resistance in outpatient samples suggests an increasing convergence between community- and healthcare-associated resistance, a phenomenon widely reported and associated with growing challenges in empirical treatment selection, particularly for urinary tract infections

From a modeling perspective, the observed limited discriminative ability (AUC = 0.62) and low explanatory power (pseudo-R² < 0.05) are consistent with the use of secondary surveillance data lacking detailed clinical variables. These findings indicate that the occurrence of the presumptive ESBL phenotype is influenced by complex and multifactorial determinants not fully captured in the available dataset, rather than reflecting poor model performance per se. Similar observations have been reported in epidemiological studies based on routine surveillance systems, where the absence of clinical, behavioral, and treatment-related variables limits predictive performance but does not compromise the identification of relevant associations.

From a public health perspective, these findings emphasize the need to strengthen regional antimicrobial resistance surveillance systems and to integrate microbiological data with clinical and structural health system indicators. Such integration is essential to inform context-specific interventions, optimize antimicrobial stewardship strategies, and improve infection prevention policies. Continued investment in surveillance infrastructure, along with the gradual incorporation of molecular resistance monitoring, represents a critical component of national and global strategies to address antimicrobial resistance [16,17]. Although phenotypic detection may underestimate underlying molecular diversity, it remains a fundamental tool for population-level surveillance in resource-limited settings.

These findings should be interpreted considering several limitations inherent to the study design and data source**.** First, the retrospective nature of the study, based on secondary microbiological surveillance data, may be subject to selection bias as well as limitations in data quality and completeness. Second, the definition of the presumptive extended-spectrum β-lactamase (ESBL) phenotype was based on phenotypic screening criteria derived from resistance to third-generation cephalosporins without confirmatory testing. Although this approach is consistent with large-scale antimicrobial resistance surveillance systems, it may have introduced misclassification bias by including other resistance mechanisms, such as AmpC β-lactamases, potentially leading to non-differential misclassification and affecting the accuracy of ESBL frequency estimates**.** Additionally, the database did not include key clinical variables such as comorbidities, prior antibiotic exposure, clinical diagnoses, or length of hospital stay. The absence of these variables limits the ability to control for residual confounding and restricts causal interpretation, as the analysis was based exclusively on microbiological and structural variables available in the surveillance system. From an analytical perspective, the limited discriminative ability (AUC = 0.62) and low explanatory power (pseudo-R² < 0.05) are consistent with the use of secondary surveillance data lacking detailed clinical information. These findings suggest that the occurrence of the presumptive ESBL phenotype is influenced by complex and multifactorial determinants not fully captured in the available dataset.

Furthermore, the multivariable regression model did not account for clustering at the hospital level. Although isolates were deduplicated to ensure one observation per patient, patients were nested within healthcare institutions, and therefore observations may not be fully independent. Individuals within the same hospital are likely to share similar clinical practices, antimicrobial use patterns, and infection control measures. This lack of adjustment may have influenced variance estimates, potentially resulting in underestimated standard errors and, consequently, an overestimation of the precision of the associations observed.

Despite these limitations, the use of a large, multicenter surveillance dataset provides a robust epidemiological framework for identifying resistance patterns and associated factors at the population level, particularly in settings where integration of clinical and microbiological data remains limited. Future studies should incorporate patient-level clinical variables, apply confirmatory ESBL testing, and consider hierarchical or multilevel modeling approaches to improve internal validity and the accuracy of effect estimates.

In conclusion, *Klebsiella pneumoniae* isolates from hospitals in the Meta Department exhibit a high and increasing prevalence of the presumptive extended-spectrum β-lactamase (ESBL) phenotype, with evidence of temporal escalation in recent years. Independent associations identified in the multivariable analysis highlight the multifactorial nature of antimicrobial resistance in this setting, rather than a single dominant determinant. These findings underscore the importance of strengthening regional surveillance systems, reinforcing antimicrobial stewardship programs, and integrating microbiological, clinical, and molecular data to better characterize resistance dynamics. Such efforts are essential to support evidence-based decision-making and to design targeted interventions aimed at mitigating the spread of antimicrobial resistance in both healthcare and community settings.
